# Glycemic management is inversely related to skeletal muscle microvascular endothelial function in patients with type 2 diabetes

**DOI:** 10.14814/phy2.14764

**Published:** 2021-03-04

**Authors:** Joshua M. Bock, William E. Hughes, Kenichi Ueda, Andrew J. Feider, Satoshi Hanada, Darren P. Casey

**Affiliations:** ^1^ Department of Physical Therapy & Rehabilitation Science Carver College of Medicine University of Iowa Iowa City IA USA; ^2^ Department of Anesthesia Carver College of Medicine University of Iowa Iowa City IA USA; ^3^ Abboud Cardiovascular Research Center Carver College of Medicine University of Iowa Iowa City IA USA; ^4^ Fraternal Order of Eagles Diabetes Research Center Carver College of Medicine University of Iowa Iowa City IA USA

**Keywords:** diabetes mellitus, endothelial function, vasodilation

## Abstract

Microvascular endothelial dysfunction precipitates cardiovascular disease mortality in patients with type 2 diabetes mellitus (T2DM). However, the relationship between glycemic management and microvascular endothelial function of these patients remains ill defined. We investigated the association between skeletal muscle microvascular endothelial function with glycemic management (HbA1c) and responses to an oral glucose challenge (OGTT) in 30 patients with T2DM (59 ± 9 years, 31.2 ± 5.1 kg/m^2^, HbA1c = 7.3 ± 1.3%). On study day 1, microvascular endothelial function was quantified as the increase (Δ from rest) in forearm vascular conductance (FVC, ml/min/100 mmHg) during intra‐arterial acetylcholine infusion at 4.0 and 8.0 μg/dl forearm volume/min (ACh4 and ACh8, respectively). [Glucose] and [insulin] were measured in a fasted state as well as following a 75 g OGTT on a second day with an additional fasting blood sample collected to measure HbA1c. FVC increased (Δ) 221 ± 118 and 251 ± 144 ml/min/100 mm Hg during ACh4 and ACh8 trials, respectively (*p* < 0.05 between doses). [Glucose] and [insulin] increased at the 1‐h time point, relative to fasting levels, and remained elevated 2 h post‐consumption (*p* < 0.05 for both variables and time points). [Glucose] nor [insulin], fasting or during the OGTT, were associated with ΔFVC during ACh4 or ACh8, respectively (*p* = 0.11–0.86), although HbA1c was inversely related (*r* = −0.47 and −0.46, respectively, *p* < 0.01 for both). Patients whose HbA1c met the ADA’s therapeutic target of ≤7.0% had greater ΔFVC to ACh4 (272 ± 147 vs. 182 ± 74 ml/100 mm Hg/min) and ACh8 (324 ± 171 vs. 196 ± 90 ml/100 mm Hg/min, *p* < 0.05 for both trials) compared to those with >7.0%, respectively. Our data show glycemic management is related to acetylcholine‐mediated vasodilation (e.g., microvascular endothelial function) in skeletal muscle of patients with T2DM.

## Graphical abstract

Data in the present study are the first to report an inverse relationship between skeletal muscle microvascular endothelial function and glycemic management (e.g., HbA1c) in patients with type 2 diabetes mellitus (T2DM). Moreover, we found patients who meet the American Diabetes Association's therapeutic target of ≤7.0% HbA1c had greater microvascular endothelial function compared to those >7.0%, yet similar to non‐diabetic controls of similar age, gender, and body mass index. Collectively, we found adequate glycemic management may offset the deleterious effects of diabetes‐induced microvascular dysfunction.

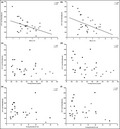

## 1 INTRODUCTION

The vascular endothelium plays a critical role in regulating perfusion in both conduit (e.g., brachial artery) and resistance vessels (Casey & Joyner, 2011). Several substances released from endothelial cells modulate vascular smooth muscle tone; however, nitric oxide (NO) is among the most relevant to vascular health (Godo & Shimokawa, 2017). Synthesized via metabolism of L‐arginine by endothelial nitric oxide synthase (eNOS), NO diffuses through the intima to neighboring smooth muscle cells elevating cyclic guanine monophosphate levels, inhibiting myosin light chain phosphatase cross‐bridge cycling, and ultimately promotes vasorelaxation (Etter et al., 2001). Indeed, eNOS‐derived NO production (endothelial function) can be assessed by stimulating calcium–calmodulin binding physically (e.g., shear stress) (Gardiner et al., 1990) and pharmacologically (e.g., acetylcholine) (Al‐Badri et al., 2019). While similar in that both produce NO, the signaling pathways of shear stress and acetylcholine are distinctly different. Here, the former relies upon mechanosensors for transmission of physical to biological signals (Davies, 2009) while the latter is contingent upon binding to muscarinic receptors (Félétou et al., 2010); nevertheless, utilizing either is an accepted means to assess vascular endothelial function in humans (Limberg et al., 2020).

Reduced ability of eNOS to produce NO, referred to as endothelial dysfunction, is a characteristic of several clinical conditions including type 2 diabetes mellitus (T2DM). Indeed, the approximate 400 million patients with T2DM (Zheng et al., 2018) are at nearly twofold greater risk of developing cardiovascular disease (CVD) in their lifetime largely attributable to endothelial dysfunction (Avogaro et al., 2006). Mechanistically, the presence of competitive substrate agonists (e.g., asymmetric dimethyl L‐arginine) (Das et al., 2011), reactive oxygen species (e.g., superoxide) (Panigrahy et al., 2017), or advanced glycation end products (Vlassara & Uribarri, 2014) attenuated NO bioavailability that promotes a pathologic milieu. Recently, Al‐Badri et al. (2019) found acetylcholine‐mediated vasodilation in the peripheral microvasculature was directly associated with observations in coronary vessels illustrating endothelial function assessed in peripheral circulation, which is considerably safer than cardiac catheterizations, provides insight to coronary vascular health.

While non‐invasive techniques are used to quantify endothelial function *in vivo* (Thijssen et al., 2011), intra‐arterial acetylcholine is particularly reflective of the microvasculature (Limberg et al., 2020). Interestingly, previous evidence using intra‐arterial acetylcholine infusion reported patients with T2DM have attenuated endothelial function (Watts et al., 1996; Williams et al., 1996); surprisingly, neither study showed a relationship between endothelial function and glycemic management. Since the mid‐1990s, significant advances in pharmacological management strategies of T2DM have been made; namely Metformin (Bailey, 2017) and novel insulin analogs (Quianzon & Cheikh, 2012). Additionally, the American Diabetes Association (ADA) published a therapeutic target of ≤7.0% HbA1c (American Diabetes Association, 2019) for patients with T2DM in 2018. Taken together, the relationship between glycemic management and microvascular endothelial dysfunction in skeletal muscle warrants revisiting. Thus, *Aim 1* sought to determine if there was a relationship between microvascular endothelial function in skeletal muscle and glycemic management (HbA1c) or responsiveness to an oral glucose tolerance test (OGTT) in patients with T2DM. *Aim 2a* was elucidated if patients with T2DM who meets the ADA’s glycemic target (≤7.0% HbA1c) (American Diabetes Association, 2019) had greater microvascular endothelial function compared to patients above this threshold. An additional objective of this *Aim* (*2b*) was to determine if those patients with an HbA1c < 7.0% had similar microvascular endothelial function compared to non‐diabetic subjects of similar age and body mass.

## 2 METHODS

### 2.1 Experimental overview

Thirty patients with T2DM and 14 non‐diabetic controls of similar age and body mass index provided written, informed consent prior to enrolment. Exclusion criteria for all subjects included symptomatic coronary artery disease, heart failure, uncontrolled hypertension, renal impairment, and current tobacco use or recent (<6 months) cessation. All but one female subject reported postmenopausal status with no hormone therapy at the time of their experimental visits. The pre‐menopausal female was using a long‐acting implantable contraceptive (Nexplanon) during her visits and had been doing so for 2 years prior to participating in the study. Experiments were performed during two separate visits separated by no more than 7 days, both in the morning after an overnight fast with subjects refraining from prescription medication use the morning of study visits as well as exercise, vitamins, supplements, alcohol, and caffeine 24 h before data collection. Vasodilatory responsiveness to intra‐arterial acetylcholine infusion, measured via Doppler ultrasound, was used to assess skeletal muscle microvascular endothelial function during the first visit. Acetylcholine was infused for 2 min at 4.0 and 8.0 μg/dl forearm volume/min with 10 min separating trials (ACh4 and ACh8, respectively). During their second visit, subjects completed an OGTT, consisting of venous blood sampling and consumption of a 75 g glucose beverage (Thermo Fisher Scientific, Waltham, MA). Study protocols were approved by the Institutional Review Board at the University of Iowa. Data in this manuscript were collected, in part of a clinical trial (ClinicalTrials.gov: NCT02804932) with some previously published findings (Bock et al., 2020).

### 2.2 Catheterization

On visit 1, a 4.5 cm, 20 g indwelling arterial catheter (RA‐04020, ARROW International) was inserted into the brachial artery under local anesthesia (1% lidocaine) and attached to a three‐port connector infused with normal saline (3 ml/h). One port was connected to a pressure transducer (model PX600F, Edwards Lifescience) positioned at heart level measuring beat‐by‐beat mean arterial pressure (MAP), and another for acetylcholine infusions. For visit 2, a 4.8 cm, 20 g catheter (BD Insyte Autogard, Beckton+Dickinson Infusion Therapy Systems Inc.) cannulated a vein, permitting blood samples throughout the study visit.

### 2.3 Vascular measurements

During visit 1, brachial artery diameter and blood velocity were measured using a Doppler probe (M12L, Vivid 7, General Electric, Milwaukee, WI) with an isonation angle of 60°. End‐diastolic diameters were obtained during the final 30 s of baselines, ACh4, and ACh8. Velocity signals were synchronized to a data acquisition system (WinDaq, DATAQ Instruments) via audio transformer (Herr et al., 2010). Forearm blood flow (FBF, cross‐sectional area times mean blood velocity) was divided by MAP to determine forearm vascular conductance (FVC, ml/min/100 mm Hg). Data were sampled at 250 Hz, stored on a computer, and analyzed offline using signal processing software (WinDaq).

### 2.4 Venous blood analysis

Samples were obtained fasted, 1 and 2 h post‐glucose consumption, and then analyzed for [glucose] and [insulin] by the University of Iowa Hospitals and Clinics. An additional fasting sample was taken for determination of HbA1c. Blood [glucose] and [insulin] responsiveness during the OGTT were quantified as the area under the time curve (AUC) as follows:

$$\frac{1\text{hr}‐\text{Fasted}}{2}+\frac{\left(1\text{hr}‐\text{Fasted}\right)+\left(2\text{hr}‐\text{Fasted}\right)}{2}$$

Insulin sensitivity was also estimated using the Matsuda Index (DeFronzo & Matsuda, 2010): $\frac{10,000}{\sqrt{{\text{Glucose}}_{0}\times {\text{Insulin}}_{0}\times {\text{Glucose}}_{120}\times {\text{Insulin}}_{120}}}$ where subscripts indicate the time of collection. Indices calculated using these time points strongly correlated with the original 5‐point equation (DeFronzo & Matsuda, 2010).

### 2.5 Statistical analysis

Data are presented as mean ± SD throughout the manuscript. Relationships between vascular responses to intra‐arterial acetylcholine infusions (e.g., ΔFVC from baseline to final 30 s of infusion) were compared with biomarkers (e.g., HbA1c) using a Pearson correlation (*Aim 1*). Independent‐samples t‐tests compared vascular responses to acetylcholine between groups (≤7.0% and >7.0% HbA1c) per trial (*Aim 2a*). If *Aim 2a* was supported, additional independent samples t‐tests were used to compare patients whose HbA1c was ≤7.0% to non‐diabetic control subjects. Demographic data were compared using independent‐samples t‐tests or chi‐squared test when appropriate. Blood [glucose] and [insulin] during the OGTT were compared across time points and between groups using a two‐way analysis of variance. All analyses were performed using SigmaPlot 11.0 software (Systat Software Inc.) with significance set a priori at *p* ≤ 0.05.

## 3 RESULTS

### 3.1 Aim 1

**FIGURE 1:**
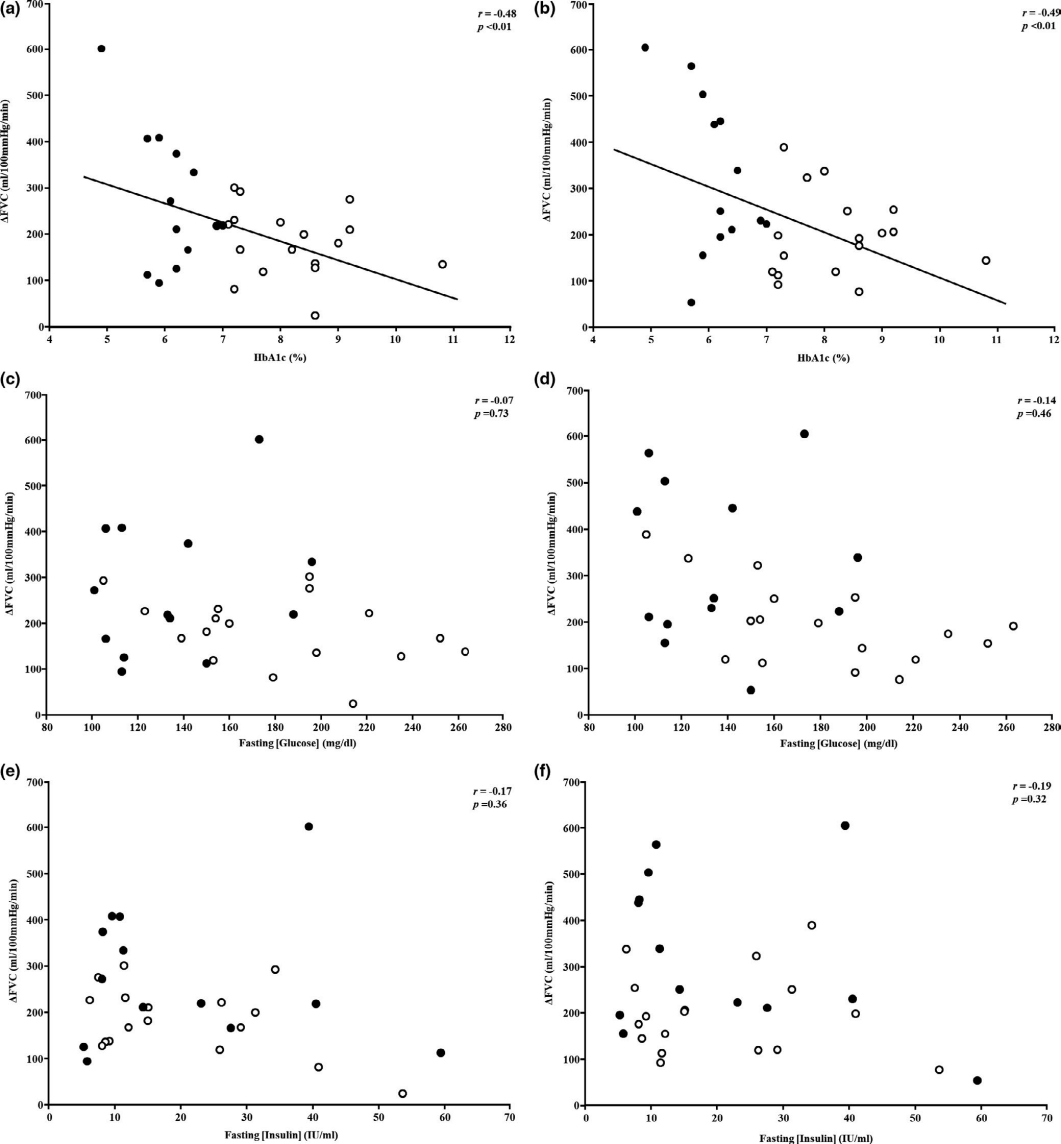
Scatter plots showing increases in forearm vascular conductance (FVC, i.e., microvascular endothelial function) during acetylcholine infusion at 4.0 (left column) and 8.0 μg/dl forearm volume/min (right column) and HbA1c (a, b), fasting blood [glucose] (c, d), as well as fasting blood [insulin] (e, f)

**FIGURE 2:**
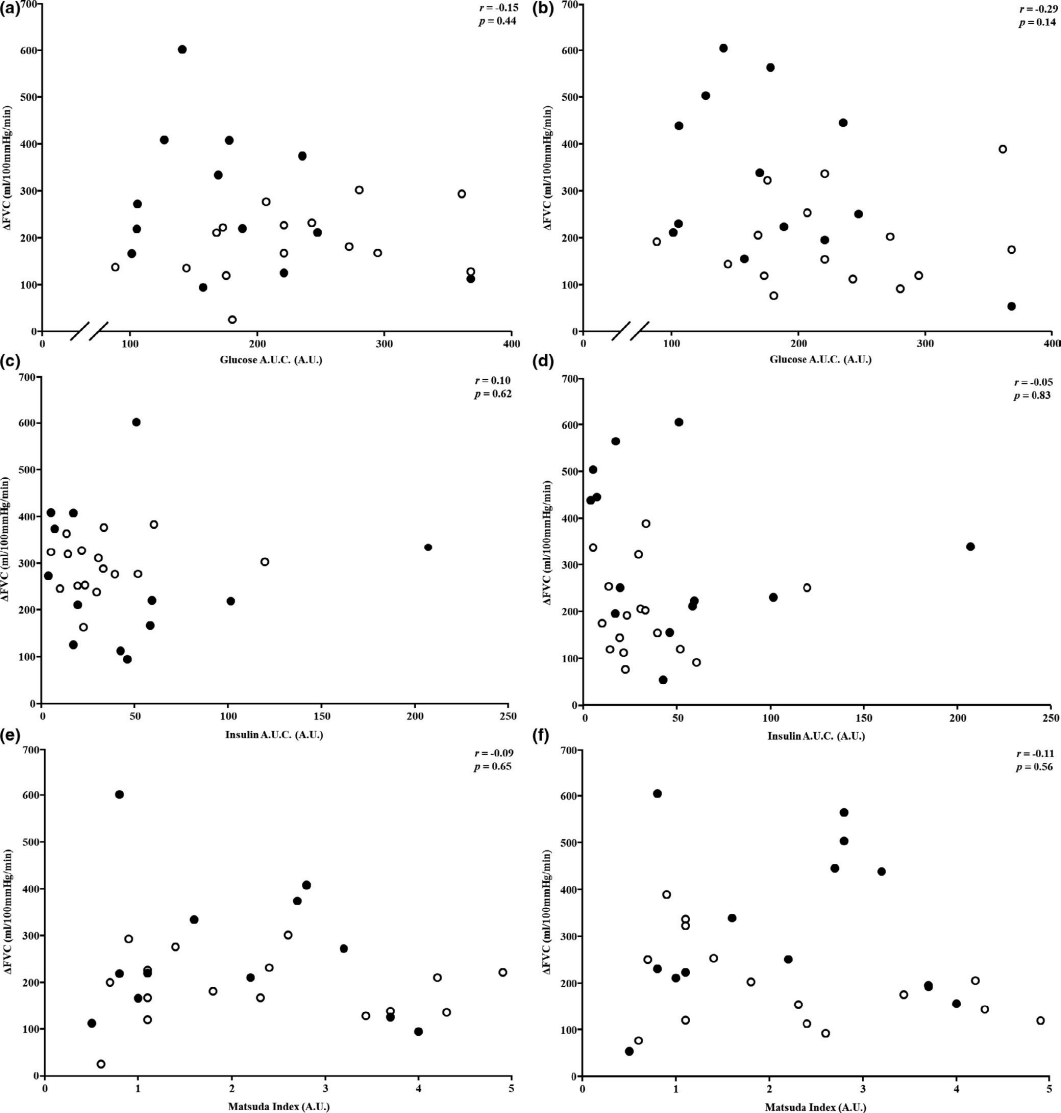
Scatter plots showing increases in forearm vascular conductance (FVC, i.e., microvascular endothelial function) during acetylcholine infusion at 4.0 (left column) and 8.0 μg/dl forearm volume/min (right column) relative to glucose (a, b) and insulin (c, d) responses (area under the curve, AUC) during an oral glucose tolerance test as well as the Matsuda Index (e, f)

Demographical data for all patients with T2DM are shown in Table 1. For the entire group of patients with T2DM, FVC increased 221 ± 118 ml/min/100 mm Hg (from 52 ± 23 to 273 ± 125 ml/min/100 mm Hg) during ACh4, whereas a 251 ± 144 ml/min/100 mm Hg increase (from 53 ± 32 to 305 ± 149 ml/min/100 mm Hg, *p* < 0.01 vs. baseline for both trials) was observed during ACh8. In the composite group of patients with T2DM, blood [glucose] increased at the 1 h (297 ± 65 mg/dl) and remained elevated 2 h (294 ± 84 mg/dl) post‐consumption, relative to fasting concentrations (162 ± 46 mg/dl, *p* < 0.05 for both time points). Likewise, [insulin] was higher 1 h (59.6 ± 48.3 μU/ml) and 2 h post‐consumption (62.7 ± 50.1 μU/ml) relative to baseline (20.3 ± 14.9 μU/ml, *p* < 0.05 for both time points). Data shown in Figure 1 illustrate associations between fasting biomarkers (HbA1c, [glucose], and [insulin]) and the vasodilatory (ΔFVC) responses during ACh4 and ACh8. HbA1c was inversely associated with ΔFVC assessed during ACh4 and ACh8 trials (*p* < 0.01 for both); however, no associations were observed for fasting [glucose] (*p* = 0.73 and 0.46) or [insulin] (*p* = 0.36 and 0.32). Scatter plots in Figure 2 represent variables collected during the OGTT and their relationship to ΔFVC responses during both ACh4 and ACh8 trials. Neither [glucose] (*p* = 0.44 and 0.14) nor [insulin] (*p* = 0.62 and 0.83) responsiveness (A.U.C.) to the OGTT or Matsuda Index (*p* = 0.65 and 0.56) correlated with microvascular endothelial function (ACh4 and ACh8 trials, respectively).

**TABLE 1:** Subject demographics for *Aim 1*

	All patients with T2DM *n* = 30
Age, years	59 ± 9
Years with T2DM	6 ± 4
Gender, F (%)	8 (27)
BMI, kg/m^2^	31.2 ± 5.1
VO_2max_, ml/kg/min	20.8 ± 6.2
Resting SBP, mm Hg	146 ± 17
Resting DBP, mm Hg	76 ± 9
HbA1c, %	7.3 ± 1.3
Fasting blood [glucose], mg/dl	162 ± 46
Fasting blood [insulin], μU/ml	20.3 ± 14.9
Matsuda index, A. U.	2.2 ± 1.3
HOMA‐IR, A. U.	0.82 ± 0.57
Prescription medications, *n* (%)	
Metformin	25 (83)
GLP‐1	4 (13)
Sulfonylurea	11 (37)
Insulin	8 (27)
Statin	20 (67)
ARB	8 (27)
ACEi	8 (27)
HCTZ	4 (13)

Note

Data are presented as mean ± SD unless otherwise noted.

Abbreviations: ACEi, angiotensin converting enzyme inhibitor; ARB, angiotensin receptor antagonist; BMI, body mass index; DBP, diastolic blood pressure; GLP‐1, glucagon‐like peptide agonist; HbA1c, glycosylated hemoglobin; HCTZ, hydrochlorothiazide; HOMA‐IR, homeostatic model assessment of insulin resistance; SBP, systolic blood pressure; T2DM, type 2 diabetes mellitus.

### 3.2 Aim 2

#### 3.2.1 Subjects

For the sub‐analyses (*Aim 2*), demographical data are shown in Table 2. There were no differences in age (*p* = 0.44), body mass index (*p* = 0.70), or resting systolic (*p* = 0.29) or diastolic (*p* = 0.29) blood pressure between groups (≤7.0% and >7.0% HbA1c). Both HbA1c and fasting blood [glucose] were greater in the T2DM cohort whose HbA1c was >7.0% relative to the ≤7.0% group (*p* < 0.01 for both) while fasting blood [insulin] tended to be higher in the former (*p* = 0.07). Groups had similar prevalence of diagnosed hypertension (*p* = 0.55) and hyperlipidemia (*p* = 0.58) as well as comparable prescribed medications (*p* = 0.06–0.23). Patients with an HbA1c > 7.0% had greater blood [glucose] pre‐glucose consumption (136 ± 32 vs. 182 ± 45 mg/dl) as well as 1 h (268 ± 64 vs. 319 ± 58 mg/dl) and 2 h (233 ± 79 vs. 343 ± 49 mg/dl) post‐consumption relative to those with ≤7.0% HbA1c, respectively (*p* < 0.05 for all). However, [insulin] did not differ between subgroups during the OGTT before (20.3 ± 16.9 vs. 20.4 ± 13.8 μU/ml, *p* = 0.49) or 1 h (64.2 ± 61.9 vs. 56.1 ± 36.4 μU/ml, *p* = 0.33) or 2 h (74.3 ± 60.9 vs. 53.3 ± 38.8 μU/ml, *p* = 0.13) post‐consumption. Similarly, there were no between‐group differences in subjects’ Matsuda index (2.4 ± 1.3 vs. 2.0 ± 1.3 for ≤7.0 and >7.0% HbA1c, respectively, *p* = 0.21). The 14 subjects without T2DM did not differ in age (*p* = 0.54), body mass index, (*p* = 0.54) or systolic (*p* = 0.87) or diastolic (*p* = 0.43) blood pressure but had lower HbA1c (*p* < 0.01) compared to patients with T2DM whose HbA1c was ≤7.0%.

**TABLE 2:** Subject demographics for *Aim 2*

	≤7.0% HbA1c *n* = 13	>7.0% HbA1c *n* = 17	Control *n* = 14
Age, years	57 ± 11	60 ± 8	60 ± 10
Years with T2DM	5 ± 4	7 ± 3	–
Gender, F (%)	4 (31)	4 (24)	5 (36)
BMI, kg/m^2^	30.8 ± 5.0	31.6 ± 5.3	29.7 ± 4.7
VO_2max_, ml/kg/min	22.4 ± 7.2	19.5 ± 5.0*	28.0 ± 8.9
Resting SBP, mm Hg	142 ± 16	149 ± 18	143 ± 13
Resting DBP, mm Hg	74 ± 8	77 ± 9	76 ± 7
HbA1c, %	6.1 ± 0.5*	8.2 ± 1.0*,^†^	5.3 ± 0.3
Fasting blood [glucose], mg/dl	136 ± 32*	182 ± 45*,^†^	95 ± 9
Fasting blood [insulin], μU/ml	15.7 ± 10.5	23.9 ± 17.0	15.6 ± 14.5
Matsuda index, AU	2.4 ± 1.3*	2.0 ± 1.3*	10.8 ± 1.3
HOMA‐IR, AU	0.79 ± 0.43*	0.85 ± 0.67*	1.92 ± 1.07
Prescription medications, *n* (%)			
Metformin	9 (69)	16 (94)	–
GLP‐1	0 (0)	4 (24)	–
Sulfonylurea	3 (23)	8 (47)	–
Insulin	2 (15)	6 (35)	–
Statin	7 (54)	13 (76)*	5 (36)
ARB	4 (31)*	4 (24)*	0 (0)
ACEi	3 (23)	5 (29)	2 (14)
HCTZ	2 (15)	2 (12)	0 (0)

Note

Data are presented as mean ± SD unless otherwise noted.

Abbreviations: ACEi, angiotensin converting enzyme inhibitor; ARB, angiotensin receptor antagonist; BMI, body mass index; DBP, diastolic blood pressure; GLP‐1, glucagon‐like peptide agonist; HbA1c, glycosylated hemoglobin; HCTZ, hydrochlorothiazide; HOMA‐IR, homeostatic model assessment of insulin resistance; SBP, systolic blood pressure; T2DM, type 2 diabetes mellitus.

* *p* ≤ 0.05 vs. control.

† *p* < 0.05 vs. ≤7.0% HbA1c.

#### 3.2.2 Forearm hemodynamics

**FIGURE 3:**
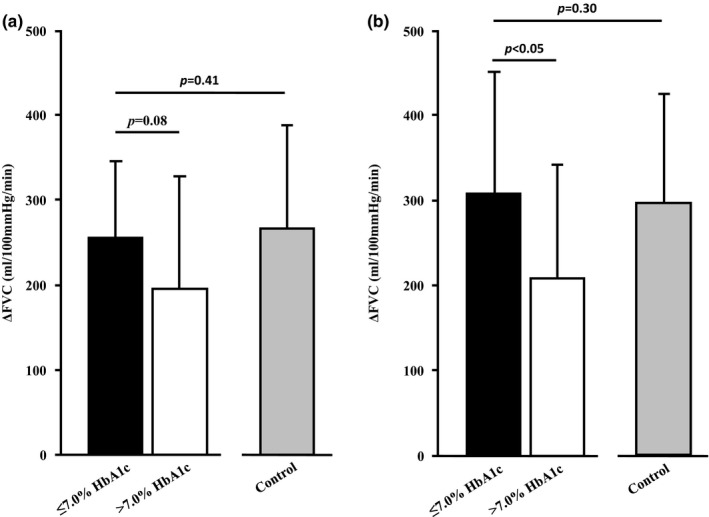
Increases in forearm vascular conductance (FVC, i.e., microvascular endothelial function) during acetylcholine infusion at 4.0 (a) and 8.0 μg/dl forearm volume/min (b). Data represent patients with type 2 diabetes mellitus whose hemoglobin glycosylation does (darker bars, *n* = 13) or does not (empty bars, *n* = 17) meet the American Diabetes Association's therapeutic target of ≤7.0% and are presented as mean ± SD. Gray bars (*n* = 14) represent non‐diabetic control subjects

Baseline ACh4 FBF (48 ± 20 vs. 57 ± 26 ml/min, *p* = 0.18), MAP (104 ± 10 vs. 102 ± 10 mm Hg, *p* = 0.31), and FVC (47 ± 21 vs. 55 ± 25 ml/100 mm Hg/min, *p* = 0.18) were similar between ≤7.0% and >7.0% HbA1c groups, respectively. During steady‐state infusion, FBF (304 ± 96 vs. 252 ± 146 ml/min, *p* = 0.14), MAP (100 ± 10 vs. 101 ± 10 mm Hg, *p* = 0.41), and FVC (303 ± 95 vs. 250 ± 143 ml/100 mm Hg/min, *p* = 0.13) were similar between T2DM subgroups. However, patients whose HbA1c was ≤7.0% tended to have greater ΔFVC during ACh4 than those with an HbA1c > 7.0% (*p* = 0.08, Figure 3a). Interestingly, the increase (Δ) in FVC in response to ACh4 was similar between the ≤7.0%HbA1c group and non‐diabetic controls (*p* = 0.41, Figure 3a). Prior to ACh8, FBF (58 ± 38 vs. 52 ± 25 ml/min, *p* = 0.28), MAP (105 ± 11 vs. 102 ± 9 mm Hg, *p* = 0.19), and FVC (56 ± 40 vs. 51 ± 25 ml/100 mm Hg/min, *p* = 0.32) were comparable between ≤7.0% and >7.0% HbA1c groups, respectively. During ACh8, FBF (364 ± 135 vs. 262 ± 139 ml/min) and FVC (363 ± 147 vs. 260 ± 138 ml/100 mm Hg/min, *p* < 0.05 for both) were greater in patients whose HbA1c was ≤7.0% compared with those >7.0%, respectively, with similar MAP (102 ± 11 vs. 101 ± 10 mm Hg, *p* = 0.42). Accordingly, patients whose HbA1c was ≤7.0% had greater ΔFVC during ACh8 relative to those >7.0% (*p* < 0.05, Figure 3b). As with ACh4, ΔFVC was similar between patients with an HbA1c ≤ 7.0% and non‐diabetic control subjects (*p* = 0.30, Figure 3b).

## 4 DISCUSSION

Principally, our findings indicate that there is no relationship between fasting [glucose] or [insulin], or the responsiveness of each variable to an OGTT, with skeletal muscle microvascular endothelial function; however, we report for the first time a moderate correlation between HbA1c and skeletal muscle microvascular endothelial function in patients with T2DM (Figures 1 and 2). Furthermore, data in Figure 3 show patients meeting the ADA’s therapeutic target of ≤7.0% HbA1c (American Diabetes Association, 2019) have greater microvascular endothelial function than patients exceeding this metric. Interestingly, patients whose HbA1c was ≤7.0% had similar microvascular endothelial function as adults free of T2DM of age, gender, and body mass index. Collectively, these data show adequate glycemic management in patients with T2DM may offset microvascular endothelial dysfunction in skeletal muscle.

Despite the novelty of our data, we are not the first to report relationships between HbA1c and endothelial function in patients with T2DM. Cross‐sectional work from Kotb et al. (Kotb et al., 2012) illustrated a strong correlation between brachial artery FMD and HbA1c in patients with T2DM along with reporting patients whose HbA1c was <7.0% had a greater FMD than those >7.0%. When data from Kotb and colleagues (Kotb et al., 2012) are compared to those from the present study, technique‐oriented differences are worth noting. While evidence supports the clinical utility of FMD (Chan et al., 2003; Gokce et al., 2003), it measures conduit artery endothelial function, whereas acetylcholine infused distal to the ultrasound probe minimizes direct effects on brachial artery diameter. Thus, vasodilatory responses shown in the present study are primarily driven by nitric oxide–mediated vasodilation (Schrage et al., 2005) within the microvasculature.

Clinically, microvascular endothelial dysfunction purportedly causes 25% of CVD‐related deaths in patients with T2DM (Jager et al., 2006). Indeed, every 1% increase in HbA1c is associated with a commensurate 17% rise in CVD‐related complications (Andersson et al., 2012). While HbA1c is a diagnostic criterion for T2DM, it also indexes glycemic management; that is, reducing HbA1c to 7.0% attenuates short‐ (AAA, 1998) and long‐term (Holman et al., 2008) risk of microvascular complications in patients with T2DM. Regarding the latter, observational findings from a 10‐year follow up on patients in the United Kingdom Prospective Diabetes Study (UKPDS) revealed those receiving intensive glycemic treatment had 13% lower incidence of myocardial infarction with commensurate 27% reduction in all‐cause mortality relative to those receiving conservative treatment (Holman et al., 2008). Our data compliment these studies by showing patients with the highest HbA1c (worse glycemic management) had the lowest vasodilatory responses to acetylcholine (worse microvascular endothelial function) in skeletal muscle (Figure 1). Further illustrating this point, patients whose HbA1c was ≤7.0% had more pronounced responses to acetylcholine infusion (better endothelial function) compared to those above the target (worse glycemic management, Figure 3). With this in mind, we should note that augmenting intensity of pharmaceutical interventions leads to diminished benefits (Adler et al., 2000) and may increase mortality rate in some patients as reported during the Action to Control Cardiovascular Risk in Diabetes (ACCORD) trial (Action to Control Cardiovascular Risk in Diabetes Study Group et al., 2008).

Findings from our data, in concert with Kotb et al. (2012), contrast work from the late 1990’s (Watts et al., 1996; Williams et al., 1996) which found no association between HbA1c and endothelial function potentially due to differences in the populations studied. Here, patients studied by Williams et al. (1996) had worse glycemic management (HbA1c = 11 ± 1%) than those in the present study (7.3 ± 1.3%), suggesting they had more pronounced endothelial dysfunction which may contribute to discrepancies in conclusions. Furthermore, ~24% of their cohort smoked tobacco products, which suppresses endothelium‐dependent vasodilation (Cui et al., 2018), whereas smokers were excluded in the present study. To this point, Watts et al. (1996) also found no association between HbA1c and endothelial function in a cohort of non‐smoking patients with T2DM with similar HbA1c as those in the present study. Second to demographical differences, both works reporting no association between HbA1c and endothelial function (Watts et al., 1996; Williams et al., 1996) used changes (Δ from pre‐infusion baseline) in vascular resistance (MAP/FBF) as a surrogate of microvascular endothelial function, whereas the present study calculated vascular conductance (FBF/MAP). Superficially, this appears to be a relatively nuanced difference; although seminal experiments from Lautt (1989) show the relationship between blood flow and conductance to be near linear, vascular resistance is curvilinear at a constant pressure. This led the author to conclude when blood flow changes to a greater extent than MAP, as occurs during acetylcholine infusion, conductance is the superior index of vascular tone; a notion later confirmed by O'Leary (1991) over a spectrum of driving pressures. Taken together, differences in subject demographics may explain some discrepancies between the present study and others (Williams et al., 1996), although the use of vascular conductance in our experiments likely explains differing conclusions within the literature (Watts et al., 1996; Williams et al., 1996). Regarding the latter, we did not observe associations between HbA1c and changes in vascular resistance during either ACh4 (*r* = 0.11, *p* = 0.58) or ACh8 (*r* = −0.09, *p* = 0.64) trials.

While data in the present study present novel findings, we recognize some limitations in our work. Firstly, the cross‐sectional nature of these experiments may not translate to interventional study designs. Indeed, longitudinal works examining the effects of exercise (Qiu et al., 2018), dietary changes (Tay et al., 2018), and pharmaceuticals (Lambadiari et al., 2018) exclusively on FMD have been conducted in patients with T2DM. Additionally, we recognize 30 patients is relatively small for a cross‐sectional design. As such, we are statistically underpowered to adjust for variables such as gender, age, or body mass index in our regression model. We also recognize muscarinic receptor expression, density, or binding affinity may have confounded findings. Indeed, preclinical data suggest that the M3 muscarinic receptor expression is elevated early on in T2DM and attenuates with disease progression (Kazuyama et al., 2009). While inclusion of an atropine (muscarinic antagonist) trial would have helped control for this, recent works have shown muscarinic receptors in cutaneous microcirculation are unaffected by T2DM (Fujii et al., 2018). We also recognize differences in aerobic fitness between groups may have contributed to our findings as patients whose HbA1c was ≤7.0% tended to have lower aerobic capacity (~20%) as non‐diabetic control subjects (*p* = 0.11). However, there were no differences between T2DM cohorts (*p* = 0.52, Table 2) despite attenuated microvascular endothelial function in the >7.0% HbA1c group (Figure 3). While microvascular endothelial function improves with exercise training (Olver & Laughlin, 2016), differences in aerobic capacity do not appear to have significantly influenced our findings.

While the deleterious effects of T2DM are observed in nearly all organ systems, microvascular endothelial dysfunction is clinically relevant as it precipitates CVD in many of these patients. Data in the present study link, for the first time, glycemic management to microvascular endothelial function in skeletal muscle of patients with T2DM (Figure 1). Moreover, our data illustrate patients who meet the ADA’s therapeutic target of ≤7.0% HbA1c (American Diabetes Association, 2019) have greater microvascular endothelial function relative to those above this mark (Figure 3). In summary, findings from the present study show adequate glycemic management appears to exert beneficial effects on microvascular function in skeletal muscle, although interventional works to corroborate this notion are warranted.

## CONFLICT OF INTEREST

The authors have no conflicts of interests to disclose.

## AUTHOR CONTRIBUTIONS

D. P. C. conceived and designed the project. J. M. B., W. E. H., K. U., A. J. F., S. H., and D. P. C. performed experiments. J. M. B. and D. P. C. analyzed and interpreted data. J. M. B. prepared figures and drafted the manuscript. J. M. B., W. E. H., K. U., A. J. F., S. H., and D. P. C. edited, revised, and approved the final version of the manuscript.
